# Cost-effectiveness and readmission rates of laparoscopic vs. open surgery for colorectal cancer: evidence from the health insurance review and assessment service dataset in South Korea

**DOI:** 10.3389/fsurg.2025.1543920

**Published:** 2025-01-20

**Authors:** Sanghyun An, Sung Eun Hong, Moo Hyun Kim, Ik Yong Kim

**Affiliations:** ^1^ Department of Colorectal Surgery, Yonsei University Wonju College of Medicine, Wonju, Republic of Korea; ^2^ Review and Assessment Division, Seoul Branch Office, Health Insurance Review and Assessment Service, Seoul, Republic of Korea

**Keywords:** colorectal neoplasms, colorectal surgery, hospital costs, hospital readmission, laparoscopy

## Abstract

**Introduction:**

We aimed to compare and analyze the cost-effectiveness of laparoscopic vs. open colorectal surgery (CRS) for colorectal cancer using health insurance claims data derived from multiple institutions in South Korea as well as the differences in hospital length of stay (LOS) and 30-day readmission rates related to postoperative complications.

**Methods:**

We retrospectively reviewed the clinical data of patients who underwent curative resection for colorectal cancer between January 1, 2020 and December 31, 2022 using national health insurance claims data in South Korea. We determined the surgical approach based on the presence or absence of treatment material codes specific to laparoscopic surgery, and divided the patients into the laparoscopic-CRS (lap-CRS) and open-CRS groups.

**Results:**

A total of 34,779 patients were included [open-CRS: 3,262 patients [9.4%]; lap-CRS: 31,517 patients [90.6%]]. The mean LOS was 14.11 and 11.27 days for the open- and lap-CRS groups, respectively (*p* < 0.001). The mean medical costs were 9,163 USD and 8,963 USD in the open- and lap-CRS groups, respectively (*p* < 0.001). A total of 1,192 (3.4%) patients were readmitted within 30 days of discharge, with a rate of 5.4% (176 cases) and 3.2% (1,016 cases) in the open- and lap-CRS groups, respectively (*p* < 0.001). Open surgery, male sex, and rectal surgery were identified as factors that increased medical cost.

**Discussion:**

According to this South Korean nationwide population-based study, laparoscopic surgery demonstrated a reduction in LOS, medical costs, and readmission rates compared with open surgery in patients with colorectal cancer.

## 1 Introduction

Globally, colorectal cancer (CRC) is the third most frequently diagnosed cancer and the second leading cause of cancer-related death (1). Over the past few decades, South Korea has experienced a significant increase in CRC incidence (2). According to Health Insurance Statistics (National Health Insurance Service, 2022) in South Korea, the number of patients treated for CRC was 148,361 in 2021, an increase of 6.6% from 2017 (3). Additionally, the per capita medical cost for CRC was 4,518 USD (5.98 million KRW) in 2021, an increase of 11.6% from 2017 (3). Therefore, the socioeconomic burden of cancer is increasing annually. Particularly, in advanced cases, the likelihood of recurrence is higher, complications are more frequent, and more complex treatment strategies are required, thereby escalating the economic burden.

Surgery is the primary treatment modality for CRC. Although various treatment options, such as preoperative chemoradiation therapy, postoperative chemotherapy, targeted therapy, and immunotherapy, have been developed to improve treatment outcomes, curative resection remains the most crucial intervention. Surgical techniques are continually advancing, improving treatment outcomes for CRC. In recent decades, minimally invasive surgery (MIS) has become the primary surgical method for CRC. MIS, including laparoscopic or robotic surgery, refers to surgical techniques that limit the size of the incisions needed and minimize physical trauma to the patient compared with traditional open surgery. MISs offer advantages, such as reduced pain, cosmetic benefits from smaller incisions, shorter hospital stays, and decreased surgical site infections (SSIs), while demonstrating short- and long-term outcomes comparable to open surgery (4–8). Currently, a large proportion of colorectal surgeries in South Korea are performed using MIS techniques. In the early 2000s, the proportion of colorectal surgeries performed laparoscopically in South Korea was less than 50%; however, this has increased to >80% in recent years (9–11).

To date, numerous studies have investigated the efficacy of laparoscopic surgery, including research on its cost-effectiveness (7, 12–14). However, the costs associated with laparoscopic surgery vary by country; therefore, the findings of a single study may not apply to all countries and situations. In South Korea, to our knowledge, there have been no large-scale studies on the cost-effectiveness of laparoscopic surgery, considering both its advantages and associated costs. Furthermore, readmission due to postoperative complications is directly linked to patients’ quality of life and safety. However, no large-scale studies to our knowledge have addressed this issue.

This study aimed to evaluate the cost-effectiveness of laparoscopic colorectal surgery (CRS) (lap-CRS) by using claims data from the Health Insurance Review and Assessment Service (HIRA), which encompasses most hospitals in South Korea. Additionally, we investigated the differences in hospital length of stay (LOS) and 30-day readmission rates associated with postoperative complications.

## 2 Materials and methods

### 2.1 Participants

This population-based retrospective study utilized National Health Insurance claims data in South Korea. The study population included all patients registered with the National Health Insurance who underwent curative resection for CRC from January 1, 2020 to December 31, 2022. The included patients were treated across all types of hospitals in South Korea, ranging from low-volume to tertiary hospitals. Patients aged <18 years and those who underwent surgery for multiple synchronous CRCs, combined surgeries involving other organs, and emergency surgery were excluded from the study. Robotic surgery was not included in this study because it is classified as a non-reimbursed medical procedure in South Korea and is therefore not captured in the National Health Insurance claims data. From January 2020 to December 2022, only the claim with the earliest start date was included in the study if multiple claims were filed for the same patient.

CRC was defined according to the Central Cancer Registry disease classification chart, which includes malignant neoplasms of the colon (C18), rectosigmoid junction (C19), and rectum (C20). CRC surgeries were defined as procedures listed in the National Health Insurance Procedure Codes. The procedure fee codes identified included QA671–673, QA679, QA921–926, QA928, Q0292, Q1261–1262, Q2671–2673, Q2679, and Q2921–2928, as listed in the health insurance medical care benefit cost book. We categorized surgeries into colon and rectal surgeries based on whether rectal resection was performed. In the extracted data, surgical approaches were classified as open or laparoscopic based on the presence of the treatment material code “N0031001,” which is specifically used to bill for the material costs associated with laparoscopic procedures.

### 2.2 Available data

The claims data contained the following clinical information: sex, age (5-year intervals), main disease code, procedure fee code, Charlson Comorbidity Index (CCI), type of surgical approach (open surgery or laparoscopy), hospital LOS, medical cost, and readmission. The CCI is a method for predicting mortality by classifying or weighting comorbidities. This index was developed in 1987 by Charlson et al. (15) as a tool to predict 1-year mortality in patients and identifies 19 significant conditions, including ischemic heart disease, diabetes, and hypertension, as comorbidities to assess the risk. In this study, comorbidity data were collected for 1 year prior to the hospital admission date for surgery. The data included primary and secondary diagnoses from both inpatient and outpatient records. After gathering the comorbidity data, the CCI was calculated.

### 2.3 Study outcomes

The primary outcomes of this study were hospital LOS, medical costs, and readmission rates. Hospital LOS was assessed based on the number of days a patient was hospitalized, as recorded in the billing statement. In this study, medical costs were defined as the total amount of patient copayments and insurer (public health fund) contributions at the time of hospitalization, as recorded in the approved total amount of billing statements. Due to the unavailability of information on non-covered expenses, these costs were not included in the medical cost analysis.

Readmission was defined as admission to the same medical institution within 30 days of discharge for the same primary diagnosis. However, admissions for planned chemotherapy or radiotherapy were considered scheduled readmissions and were excluded from this analysis. For the readmitted patients, the entire list of secondary diagnoses billed at the time of readmission was reviewed. After expert consultation, specific secondary conditions that could have caused readmission were identified and classified as either surgery-related or non-surgical general complications.

### 2.4 Statistical analysis

Categorical variables were analyzed using the chi-square test and presented as frequencies and percentages. Continuous variables were analyzed using the Student's *t*-test and expressed as mean values and standard deviations. After conducting normality tests, non-normally distributed data were analyzed using the Mann–Whitney U test and were described as medians and interquartile ranges. After reflecting the adjustment variables and conducting normality tests, a multiple linear regression analysis was performed to identify factors influencing hospital LOS and medical costs. After incorporating the adjustment variables, a binary multiple logistic regression analysis was conducted to identify the factors influencing readmission.

All data analyses were conducted using SAS Enterprise Guide, version 7.1 (SAS Institute, Cary, NC, USA), and statistical significance was determined at a level of.05 with a 95% confidence interval (CI).

## 3 Results

### 3.1 Patient enrollment and baseline characteristics

**Figure 1:**
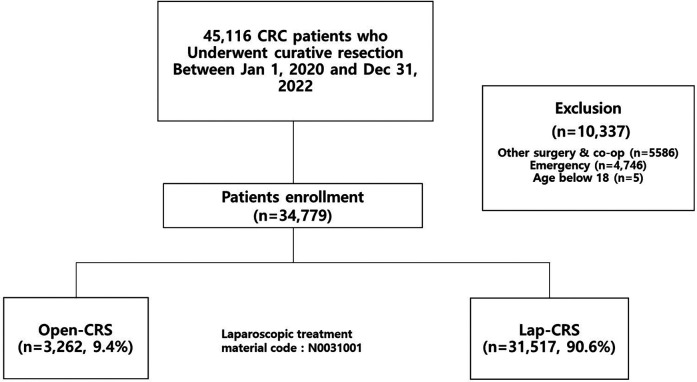
Patients enrollment.

During the study period, of 45,116 patients diagnosed with CRC and undergoing surgery, 10,337 were excluded based on our inclusion criteria, resulting in a final cohort of 34,779 patients included in the analysis (Figure 1). Among the study participants, 20,934 (60.2%) were male and 13,845 (39.8%) were female, with both the open-CRS and lap-CRS groups showing a higher proportion of males than females. The mean age of all the study participants was 65.71 years, with a mean age of 66.27 years in the open-CRS group and 65.65 years in the lap-CRS group, indicating similar age distributions between the two groups. Among all patients, the largest proportion were aged between 60 and 64 years (5,427 patients; 15.6%). In the open-CRS group, the largest subgroup consisted of patients aged ≥80 years, with 535 (16.4%) patients, whereas in the lap-CRS group, the largest subgroup consisted of patients aged between 60 and 64 years at 4,979 (15.7%) patients (Table 1).

**Table 1:** Baseline characteristics.

Variables	Total (*n* = 34,779)		Open-CRS (*n* = 3,262, 9.4%)		Lap-CRS (*n* = 31,517, 90.6%)		*p*-value
	Case	(%)	Case	(%)	Case	(%)	
Sex							0.017
Male	20,934	60.2	2,027	62.1	18,907	60.0	
Female	13,845	39.8	1,235	37.9	12,610	40.0	
Age							
Avg	65.71		66.27		65.65		<0.001
18–49	3,108	8.9	325	10	2,783	8.8	
50–54	3,163	9.1	281	8.6	2,882	9.1	
55–59	3,979	11.4	354	10.9	3,625	11.5	
60–64	5,427	15.6	480	14.7	4,947	15.7	
65–69	5,211	15	421	12.9	4,790	15.2	
70–74	4,903	14.1	422	12.9	4,481	14.2	
75–79	4,410	12.7	444	13.6	3,966	12.6	
≥80	4,578	13.2	535	16.4	4,043	12.8	
Tumor location							<0.001
Colon	13,052	37.5	1,651	50.6	11,401	36.2	
Rectum	21,727	62.5	1,611	49.4	20,116	63.8	
CCI							<0.001
0 point	5,205	15.0	411	12.6	4,794	15.2	
1 point	2,272	6.5	175	5.4	2,097	6.7	
2 point	5,713	16.4	470	14.4	5,243	16.6	
≥ 3 point	21,589	62.1	2,206	67.6	19,383	61.5	
Readmission							<0.001
Yes	1,192	3.4	176	5.4	1,016	3.2	
No	33,587	96.6	3,086	94.6	30,501	96.8	
Average medical cost (USD)	8,982.26 ± 2,783.22		9,163.09 ± 4,118.37		8,963.55 ± 2,605.65		<0.001
Average LOS (days)	11.53 ± 5.48		14.11 ± 7.88		11.27 ± 5.09		<0.001

CRS, colorectal surgery; CCI, Charlson Comorbidity Index; USD, United States dollar; LOS, length of stay.

When dividing the patient groups into colon and rectal categories, 1,611 patients (49.4%) in the open-CRS group and 20,116 (63.8%) in the lap-CRS group had rectal cancer. Laparoscopic surgery was performed in 92.6% of patients who underwent surgery for rectal cancer and 87.4% of those who underwent surgery for colon cancer. In terms of the patients’ comorbidities, 2,206 patients (67.6%) in the open-CRS group had a CCI score of ≥3, which was higher than that of the 19,383 patients (61.5%) in the lap-CRS group.

### 3.2 Hospital LOS

**Figure 2:**
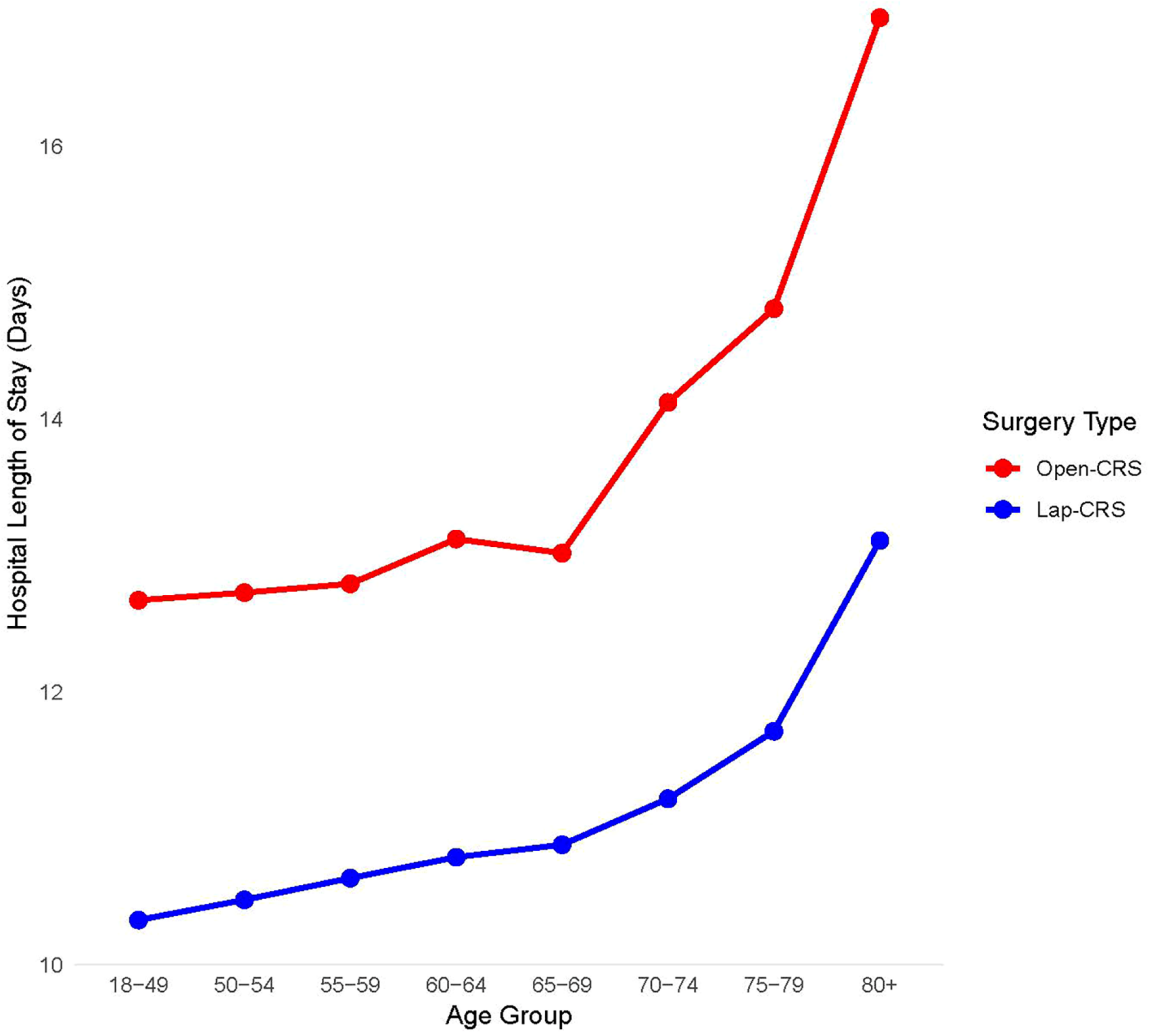
Hospital length of stay by age group and surgery type.

The average LOS was 14.11 days in the open-CRS group and 11.27 days in the lap-CRS group, indicating that the lap-CRS group had a 2.84-day shorter hospital stay than the open surgery group. This result showed the same trend regardless of age, sex, tumor location, or CCI. In both the groups, the hospital LOS increased with age. The difference in the average LOS between the open- and lap-CRS groups tended to increase with age, with the largest difference observed in patients aged ≥80 years where the lap-CRS group had a 3.91-day shorter stay (Table 2 and Figure 2).

**Table 2:** Difference of LOS (days).

Variables	Open LOS	Lap LOS	Difference	*p*-value
	Avg	Avg	Open—Lap	
Sex				
Male	14.29	11.49	2.8	<0.001
Female	13.78	10.93	2.86	<0.001
Age				
18–49	12.74	10.32	2.41	<0.001
50–54	12.54	10.53	2.02	<0.001
55–59	12.66	10.68	1.98	<0.001
60–64	13.15	10.85	2.3	<0.001
65–69	13.31	10.89	2.42	<0.001
70–74	14.73	11.29	3.44	<0.001
75–79	14.63	11.82	2.8	<0.001
≥80	17.24	13.33	3.91	<0.001
Cancer location				
Colon surgery	14.99	11.4	3.59	<0.001
Rectal surgery	13.18	11.18	2	<0.001
CCI				
0 point	14.33	10.91	3.41	<0.001
1 point	15.05	11.49	3.56	<0.001
2 point	13.39	10.97	2.42	<0.001
≥ 3 point	14.13	11.41	2.73	<0.001

LOS, length of stay; CCI, Charlson Comorbidity Index.

According to multiple linear regression analysis, male sex, age ≥60 years, rectal surgery, and a high CCI were identified as factors that increased the hospital LOS. Compared with laparoscopic surgery, open surgery was associated with a longer LOS (regression coefficient 2.65, 95% CI 2.47–2.84). Additionally, females had a shorter LOS than males (regression coefficient −0.64, 95% CI −0.75 to −0.53). The LOS was significantly longer in patients aged ≥60 than in those <50 years. Specifically, in patients aged ≥80 years, the LOS increased by approximately 2.57 days compared with those aged <50 years (95% CI 2.34–2.81). Additionally, rectal surgery was associated with a longer LOS compared with colon surgery (regression coefficient 0.96, 95% CI 0.82–1.09). It was also found that for each one-point increase in the CCI score, the LOS increased by 0.09 days (Table 3).

**Table 3:** Multiple linear regression analysis for determinants of LOS.

Variables	β	Std.Error	*p*-value
Surgical method			
Open-CRS	ref		
Lap-CRS	−2.62	0.09	<0.001
Sex			
Male	ref		
Female	−0.72	0.05	<0.001
Age			
18–49	ref		
50–54	0.11	0.14	0.438
55–59	0.22	0.13	0.104
60–64	0.31	0.12	0.013
65–69	0.36	0.13	0.004
70–74	0.75	0.13	<0.001
75–79	1.25	0.13	<0.001
≥80	2.79	0.12	<0.001
Tumor location			
Colon surgery	ref		
Rectal surgery	−0.25	0.06	<0.001
CCI	0.08	0.01	<0.001

CI, confidence interval; CRS, colorectal surgery; CCI, Charlson Comorbidity Index.

### 3.3 Medical costs

**Figure 3:**
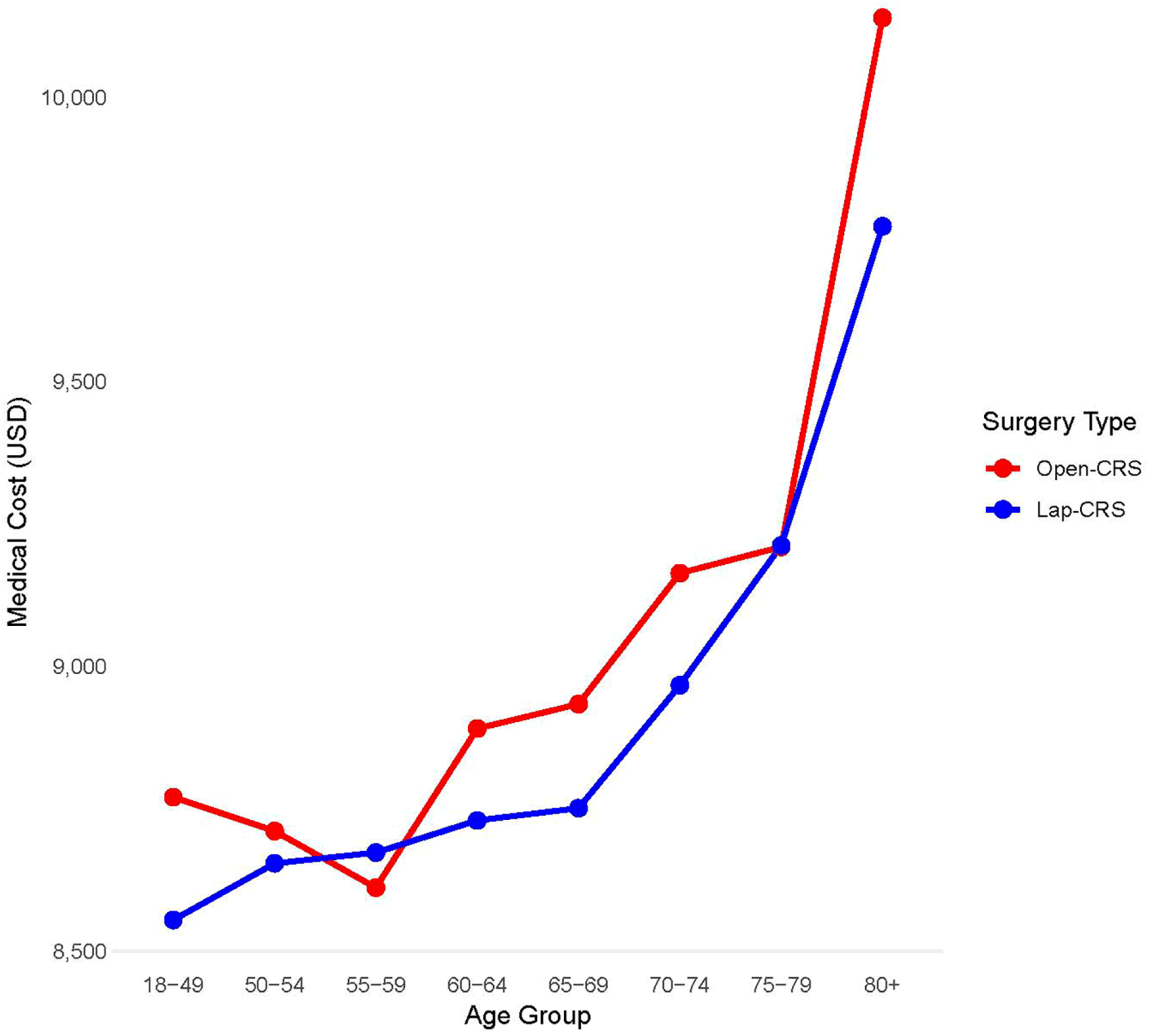
Medical costs by age group and surgery type.

The average medical costs were 9,163 USD for the open-CRS group and 8,963 USD for the lap-CRS group, with the laparoscopic surgery group incurring 199 USD less than the open surgery group. Medical costs were lower in the lap-CRS group for both males and females and increased with age in both groups. Except for the 55–59 and 75–79 years age groups, the medical costs of laparoscopic surgery were lower in most age groups. The cost difference between the two groups was largest for patients aged ≥80 years, amounting to 364 USD (Figure 3). In colon surgery cases, the lap-CRS group incurred 686 USD less than the open-CRS group; however, no significant difference was observed in rectal surgery. When comparing costs based on the CCI, only patients with a CCI score of ≥3 had lower medical costs in the lap-CRS group than that of those in the open-CRS group (Table 4).

**Table 4:** Difference in medical costs between lap-CRS and open CRS.

Variables	Open cost	Lap cost	Difference	*p*-value
	Avg	Avg	Open—Lap (USD)	
Sex				
Male	9,309.89	9,157.58	152.31	0.026
Female	8,922.16	8,672.62	249.54	0.001
Age				
18–49	8,773.42	8,555.24	218.18	0.103
50–54	8,697.85	8,660.91	36.95	0.806
55–59	8,420.02	8,674.40	−254.38	0.037
60–64	9,020.76	8,759.28	261.49	0.043
65–69	8,973.11	8,769.76	203.35	0.109
70–74	9,336.37	9,007.31	329.06	0.022
75–79	9,218.50	9,253.83	−35.33	0.812
≥80	10,230.39	9,865.87	364.52	0.029
Cancer location				
Colon surgery	9,145.72	8,459.29	686.43	<0.001
Rectal surgery	9,180.89	9,249.34	−68.45	0.303
CCI				
0 point	9,318.18	8,929.51	388.67	0.071
1 point	9,491.71	9,085.47	406.24	0.203
2 point	8,711.62	8,778.83	−67.21	0.704
≥ 3 point	9,204.32	9,008.74	195.58	0.031

CCI, Charlson Comorbidity Index.

According to multiple linear regression analysis, open surgery, male sex, age ≥65 years, rectal surgery, and a high comorbidity index were identified as factors that increased medical costs. Compared with open surgery, laparoscopic surgery reduced medical costs (regression coefficient, −220.81 USD, SE 50.21). Additionally, female patients had lower medical costs than male patients (regression coefficient, −457 USD, SE 30.10). Medical costs were significantly higher in patients aged ≥65 years than that in those aged <50 years. In particular, patients aged ≥80 years had medical costs that were approximately 1296 USD higher than those aged <50 years (SE 64.38). Medical costs were higher for rectal surgery than for colon surgery (regression coefficient, 767 USD, SE 30.63), and for each one-point increase in the CCI, medical costs increased by 41 USD (Table 5).

**Table 5:** Multiple linear regression analysis for determinants of medical costs.

Variables	β	Std. Error	*p*-value
Surgical method			
Open-CRS	ref		
Lap-CRS	−220.81	50.21	<0.001
Sex			
Male	ref		
Female	−457.24	30.10	<0.001
Age			
18–49	ref		
50–54	19.14	72.82	0.792
55–59	23.96	68.26	0.725
60–64	97.65	64.49	0.130
65–69	125.90	64.05	0.049
70–74	367.09	65.54	<0.001
75–79	634.50	66.63	<0.001
≥80	1,296.36	64.38	<0.001
Tumor location			
Colon surgery	ref		
Rectal surgery	767.37	30.63	<0.001
CCI	41.02	4.10	<0.001

CI, confidence interval; CRS, colorectal surgery.

### 3.4 Readmission

The 30-day readmission rates were 5.4% (176 cases) in the open-CRS group and 3.2% (1,016 cases) in the lap-CRS group, indicating that laparoscopic surgery had a lower readmission rate than open surgery.

Binary logistic regression analysis was performed to identify factors influencing the readmission rate after adjusting for all independent variables. Open surgery, male sex, rectal surgery, and a high CCI were identified as factors that increased the risk of readmission. Compared with laparoscopic surgery, open surgery was associated with a 1.611 times higher likelihood of readmission [odds ratio (OR) = 1.611, 95% CI = 1.362–1.905, *p* < 0.001]. Additionally, females had a 0.790 times lower likelihood of readmission than males (OR = 0.790, 95% CI = 0.698–0.895, *p* < 0.001). By age group, the likelihood of readmission was 0.737 times lower in patients aged 70–74 years than in those aged 18–49 years (OR = 0.737, 95% CI = 0.572–0.949, *p* < 0.05), while no significant relationship was found for other age groups. Patients who underwent rectal surgery had a 1.618 times higher readmission rate than those who underwent colon surgery (OR = 1.618, 95% CI = 1.385–1.890, *p* < 0.001). Additionally, for each one-point increase in the CCI, the likelihood of readmission increased 1.042 times (OR = 1.042, 95% CI = 1.027–1.057, *p* < 0.001) (Table 6).

**Table 6:** Binary logistic regression analysis for determinants of readmission.

Variables	Adjusted OR	95% CI	*p*-value
Surgical method			
Open-CRS	ref		
Lap-CRS	0.61	0.52–0.72	<0.001
Sex			
Male	ref		
Female	0.77	0.68–0.87	<0.001
Age			
18–49	ref		
50–54	0.91	0.68–1.21	0.506
55–59	0.96	0.74–1.26	0.770
60–64	0.92	0.72–1.18	0.503
65–69	0.95	0.74–1.22	0.684
70–74	0.83	0.64–1.08	0.154
75–79	0.84	0.65–1.09	0.188
≥80	0.81	0.63–1.05	0.114
Tumor location			
Colon surgery	ref		
Rectal surgery	0.92	0.81–1.03	0.154
CCI	1.04	1.02–1.06	<0.001

CI, confidence interval; CRS, colorectal surgery; CCI, Charlson Comorbidity Index.

To analyze the causes of readmission, we examined the diagnoses from the claims of 1,192 readmission cases, focusing on those with at least one primary diagnosis. Among these, 567 (11.0%) were identified as potential causes of readmission. Surgery-related complications included diagnostic codes for bowel obstruction, wound infection, and peritonitis, whereas general complications included codes for urological, thromboembolic, and respiratory complications. The most common causes were bowel obstruction (*n* = 211; 37.2%), followed by urological complications (*n* = 169; 29.8%), wound infections (*n* = 109;19.2%), peritonitis (*n* = 40; 7.1%), thromboembolic complications (*n* = 21; 3.7%), and respiratory complications (*n* = 17; 3.0%). While there were no statistically significant differences in the distribution of readmission causes between the laparoscopic and open surgery groups (*p* = 0.066), specific differences were observed in certain complications. Bowel obstruction had a lower incidence in the laparoscopic surgery group (4.0%) compared to the open surgery group (4.9%), and wound infection showed a more pronounced difference, with rates of 1.9% in the laparoscopic group vs. 3.3% in the open surgery group. When comparing the average medical costs of readmission, there was no significant difference between the open-CRS and lap-CRS groups (3,172 vs. 3,074 USD, *p* = 0.786). Similarly, the LOS during readmission did not differ significantly between the two groups (9.78 ± 8.57 days vs. 9.41 ± 8.34 days, *p* = 0.589) (Supplementary Table 1).

## 4 Discussion

This population-based study revealed that, between 2020 and 2022, approximately 90.6% of CRC surgeries in South Korea were performed laparoscopically. Furthermore, lap-CRS reduced the hospital LOS, medical costs, and 30-day readmission rate compared with open-CRS.

The hospital LOS is an important clinical indicator of a patient's postoperative recovery status. Considering the patient's quality of life and economic aspects, reducing the hospital LOS after surgery is an ancillary goal for every surgeon. In our analysis, the lap-CRS group had an average LOS of 11.27 days, which was 2.84 days shorter than that in the open-CRS group. In addition to laparoscopic surgery, female sex, younger age, and lower CCI were identified as factors associated with reduced hospital LOS. This result is consistent with those of other studies on laparoscopic surgery. In our previous study investigating the impact of laparoscopic surgery on SSIs, the laparoscopic surgery group had a significantly shorter average hospital LOS, at 12.18 days, than the open surgery group at 14 days (16). Additionally, in a study by Son et al. (5) focusing on patients with CRC aged ≥80 years, patients who underwent laparoscopic surgery had a hospital LOS that was approximately 3.6 days shorter and started a soft diet approximately 2.5 days earlier than that of those who underwent open surgery. Furthermore, the incidence of complications was significantly lower in the laparoscopic surgery group and the long-term outcomes were similar to those in the open surgery group. Studies conducted on patients who underwent CRC surgery abroad have also reported that the hospital LOS for laparoscopic surgery is shorter than that for open surgery (17). In studies from other countries, the average LOS after CRS has been reported to be approximately 10 days. Although this is slightly different from the average LOS in our study at 11.53 days, this difference can be attributed to variations in healthcare environments across countries. Furthermore, because of the nature of the claims data, our study lacked information on the exact date of surgery; therefore, we analyzed the total LOS rather than the postoperative LOS. This may have resulted in slight differences compared with other studies that have focused on postoperative LOS.

In our analysis of medical costs, open surgery was found to be 199 USD more expensive than laparoscopic surgery; after adjusting for independent variables, the cost difference was 220 USD. During the study period, the additional cost of laparoscopic surgery in South Korea was 316 USD (239,000 KRW) for laparoscopic materials, and there was no difference in procedure fees between open and laparoscopic surgeries; therefore, the billed surgical procedure fees are the same. Therefore, laparoscopic surgery typically incurs higher total surgical costs owing to the additional laparoscopic material costs. Nevertheless, laparoscopic surgery is more economical because of its various positive effects, such as a reduced LOS and decreased incidence of complications. Despite differences in healthcare costs between countries, the economic benefits of lap-CRS are consistent with those reported in several international studies. In the early stages of its introduction, studies indicated that laparoscopic surgery was costlier because of the initial adaptation to the new surgical technique. However, as laparoscopic surgery has become more widespread, its economic benefits have been recognized (7, 8, 12, 18, 19). According to a nationwide study conducted by Keller et al. (7) in the United States, the total medical cost is lower for laparoscopic than for open CRS (17,268 vs. 20,552 USD, *p* < 0.0001). Their study detailed the specific medical costs and showed that while the pure surgery cost was higher for laparoscopic surgery than for open surgery, the costs of hospital stay due to shorter LOS and other costs related to fewer complications were lower for laparoscopic surgery than for open surgery.

Early readmission after discharge is a critical patient-centered outcome with significant implications for patient safety and quality of life. Postoperative complications and unplanned readmissions have become major quality indicators for health systems, with one incident of readmission estimated to cost upwards of 9,000 USD (20). In our study, the overall readmission rate was 3.4%, with 1,192 of 34,779 patients being readmitted, with the rate being significantly lower in the lap-CRS group. According to several previous studies, the readmission rate in patients who underwent elective CRS ranges from 6% to 20% (20, 21),. In a previous study, Kim et al. (22) reported that among 457 patients who underwent low anterior resection for primary rectal cancer, 22 patients (4.8%) were readmitted within 30 days after discharge. The primary causes of readmission were postoperative intestinal obstruction due to adhesions and anastomotic leakage. Chung et al. (23) reported that of 292 patients who underwent CRC surgery, 24 (10.5%) were readmitted. The most common readmission diagnoses were wound bleeding, SSI, bowel obstruction or blockage, diarrhea, and hepatobiliary disorders. Additionally, they reported that the 5-year overall survival (OS) and disease-free survival were significantly lower in the readmission group than that in the non-readmission group. According to a population-based study conducted by Greenblatt et al. (24), the 30-day readmission rate was 11.0% among 42,348 patients who underwent colectomy. The main causes of readmission were bowel obstruction and infections. Their study found that the risk factors for 30-day readmission included male sex, comorbidities, stoma creation, and complications, which is similar to the findings of our study. Esemuede et al. (25) reported that among patients who underwent colorectal resection, those who underwent rectal resection had higher readmission rates than those who underwent colectomy and that laparoscopic surgery had lower readmission rates than open surgery. Additionally, laparoscopic surgery was associated with significantly lower rates of SSI, bleeding, reoperation, 30-day mortality, and overall complications. Similarly, our study found that the readmission rate was higher for rectal surgery than that for colon surgery (OR 1.618, 95% CI 1.385–1.890), with the most common surgery-related reasons for readmission being bowel obstruction and SSI.

Using public data, we calculated the CCI based on patient diagnoses. Higher CCI scores, indicating more underlying conditions, were associated with increased LOS, medical costs, and readmission rates. There are several tools for evaluating comorbidities, such as the Elixhauser Comorbidity Index, Cumulative Illness Rating Scale, and Adult Comorbidity Evaluation-27. Among these, the CCI offers the advantage of being simple and quick to use for predictive purposes, and its reliability and validity are well established (15). Several previous studies on patients with CRC have suggested that a high CCI score is associated with increased hospitalization costs, longer hospital LOS, and higher mortality (26, 27). Furthermore, Tominaga et al. (28) conducted a study using various prognostic markers to predict outcomes in patients with CRC aged ≥75 years. They reported that patients with a high CCI score had lower OS than those with a lower score of 0 or 1.

This study had several limitations. First, owing to the nature of the claims data, there were limitations in including detailed clinical information. We were unable to obtain information on the cancer stage, functional status, operation time, and intraoperative complications, which prevented us from considering various clinical scenarios. Second, owing to the inability to verify information on open conversion due to the inherent limitations of the public dataset, converted cases were included in the laparoscopic surgery group for analysis, which may have introduced a bias. Third, the health insurance claims data did not include information on uncovered treatments, procedures, and medications, which limited the analysis of medical costs. However, given the significant variation in uncovered treatments, procedures, and medications based on institutional and surgeon-specific practices, excluding these items from the analysis could help minimize variability and improve the consistency and generalizability of the cost analysis. Fourth, the application of Enhanced Recovery After Surgery protocols, which have been shown to reduce hospital LOS, was not reflected in this study, and this should be considered a limitation. Despite these limitations, our study had several strengths. We used nationwide data from all patients who underwent CRS in South Korea. Although the health insurance claims data lack information on non-covered treatments, procedures, and medications, they include comprehensive details of all medical services utilized by patients, excluding non-covered items. This allowed us to generalize our analysis results. Additionally, the variables analyzed in this study, such as LOS, medical costs, and readmissions, are crucial for evaluating the quality and outcomes of medical services. Our findings based on big data provide valuable insights into these aspects.

According to our nationwide Korean population-based study, laparoscopic surgery demonstrated significant advantages over open surgery in patients with CRC, including reduced medical costs, hospital LOS, and readmission rates. The lower readmission rate suggests that the economic benefits of laparoscopic surgery may be even greater than indicated by our findings when considering the costs associated with readmissions. In an era in which MIS is the standard approach, our study reinforces these benefits through big data analysis. Additionally, with the increasing use of laparoscopic surgery among elderly patients, future research should aim to verify whether these advantages also extend to older populations. These insights offer valuable guidance for healthcare providers and policymakers to enhance patient outcomes and healthcare efficiency.

## Data Availability

The raw data supporting the conclusions of this article will be made available by the authors, without undue reservation.
