# Supplementary material for: Cost-effectiveness and readmission rates of laparoscopic vs. open surgery for colorectal cancer: evidence from the health insurance review and assessment service dataset in South Korea
Source: Front Surg. 2025 Jan 20;12:1543920. doi: 10.3389/fsurg.2025.1543920 (PMC11788362; doi:10.3389/fsurg.2025.1543920)
Supplement: Supplementary file 1 [file Table1.docx]

Supplementary Material

# Supplementary Table 1. Causes of illness leading to readmission.

| Cause | Total (n=5,137) | | Open-CRS (n=695) | | Lap-CRS (n=4,442) | | *p*-value |
| --- | --- | --- | --- | --- | --- | --- | --- |
|  | Number | (%) | Number | (%) | Number | (%) |  |
| Operation-related |  |  |  |  |  |  | 0.066 |
| Ileus | 211 | 4.1 | 34 | 4.9 | 177 | 4.0 |  |
| Wound infection | 109 | 2.1 | 23 | 3.3 | 86 | 1.9 |  |
| Peritonitis | 40 | 0.8 | 4 | 0.6 | 36 | 0.8 |  |
|  |  |  |  |  |  |  |  |
| General |  |  |  |  |  |  |  |
| Urinary | 169 | 3.3 | 21 | 3.0 | 148 | 3.3 |  |
| Thromboembolic | 21 | 0.4 | 6 | 0.9 | 15 | 0.3 |  |
| Pulmonary | 17 | 0.3 | 3 | 0.4 | 14 | 0.3 |  |

CRS, colorectal surgery
